# Colorectal cancer screening methods: A systematic review

**DOI:** 10.1097/MD.0000000000044759

**Published:** 2025-10-03

**Authors:** Abdul Subhan Talpur, Abdulrahman Atasi, Corey Geoffrey Knowles, Farukhzad Hafizyar, Abdul Khan Basit, Sadaf Showkat, Asadullah Memon, Abida Parveen

**Affiliations:** a Department of Medicine, Ibn e Seena Hospital, Kabul, Afghanistan.

**Keywords:** colonoscopy, colorectal cancer, cost-effectiveness, early detection, fecal immunochemical test, risk populations, screening methods

## Abstract

**Background:**

Colorectal cancer (CRC) is a leading cause of morbidity and mortality worldwide. Early detection through screening has been proven to reduce CRC incidence and mortality. Various screening methods, including colonoscopy, fecal immunochemical test (FIT), and others, are employed with varying degrees of effectiveness and cost-efficiency. However, their performance across different risk populations remains poorly understood. This systematic review aims to compare the effectiveness and cost-efficiency of commonly used CRC screening methods (colonoscopy, FIT, and others) in various risk populations, including those at average risk, high-risk, and very high-risk (genetic predisposition, family history, etc.).

**Methods:**

A comprehensive literature search was conducted across multiple databases (PubMed, Scopus, etc.) to identify studies published between 2000 and 2024. Studies evaluating CRC screening methods in different risk populations were included. Data regarding screening effectiveness, cost-effectiveness, sensitivity, specificity, and patient compliance were extracted. Risk populations were classified as average risk, high risk (age, family history), and very high risk (genetic syndromes, inflammatory bowel disease).

**Results:**

A total of 9 studies were included in the analysis. Colonoscopy was found to be the most accurate in detecting advanced lesions but also the most expensive and invasive. FIT demonstrated good sensitivity and specificity, particularly in average-risk populations, with lower costs and greater patient compliance. Alternative methods such as CT colonography and sigmoidoscopy showed varied results in terms of effectiveness and cost-efficiency across risk groups. The cost-effectiveness analysis highlighted FIT as the most cost-efficient method for average-risk individuals, while colonoscopy remains essential for high- and very high-risk populations.

**Conclusions:**

The effectiveness and cost-efficiency of CRC screening methods vary significantly across risk populations. While colonoscopy remains the gold standard for high-risk and very high-risk individuals, FIT offers a cost-effective and non-invasive alternative for average-risk populations. Tailoring screening strategies based on individual risk profiles can optimize outcomes and resource allocation in CRC prevention programs. Further research is needed to explore the long-term cost-effectiveness and patient outcomes associated with these screening methods in different populations.

## 1. Introduction

Colorectal cancer (CRC) is a leading cause of cancer-related morbidity and mortality worldwide.^[1–5]^ Early detection through screening is the most effective strategy for reducing CRC incidence and mortality by identifying precancerous lesions and early-stage cancers. Various screening methods, such as colonoscopy, fecal immunochemical test (FIT), and newer options like CT colonography (CTC) and sigmoidoscopy, are available.^[6]^ Each method offers distinct advantages and limitations regarding effectiveness, cost-efficiency, and patient compliance. Colonoscopy is widely regarded as the gold standard for CRC screening due to its high sensitivity in detecting colorectal neoplasms, including cancers and polyps. However, it is invasive, expensive, and associated with lower patient compliance.^[7]^ On the other hand, FIT is a noninvasive and cost-effective alternative, particularly suitable for average-risk individuals. Despite its lower sensitivity, FIT offers simplicity, greater patient acceptance, and significant cost savings. Risk factors, such as age, family history, genetic predisposition, and inflammatory bowel disease, play a key role in determining the most appropriate screening method.^[8]^ High-risk individuals often require more intensive screening, including early colonoscopy, while average-risk individuals may benefit from less invasive options like FIT. However, there is a lack of comprehensive studies comparing the effectiveness and cost-efficiency of these screening methods across different risk populations. This systematic review aims to compare the effectiveness and cost-efficiency of various CRC screening methods, such as colonoscopy, FIT, and others, in different risk populations to provide evidence-based recommendations for optimal, personalized screening strategies.

## 2. Methods

### 2.1. Study design

**Figure 1.**
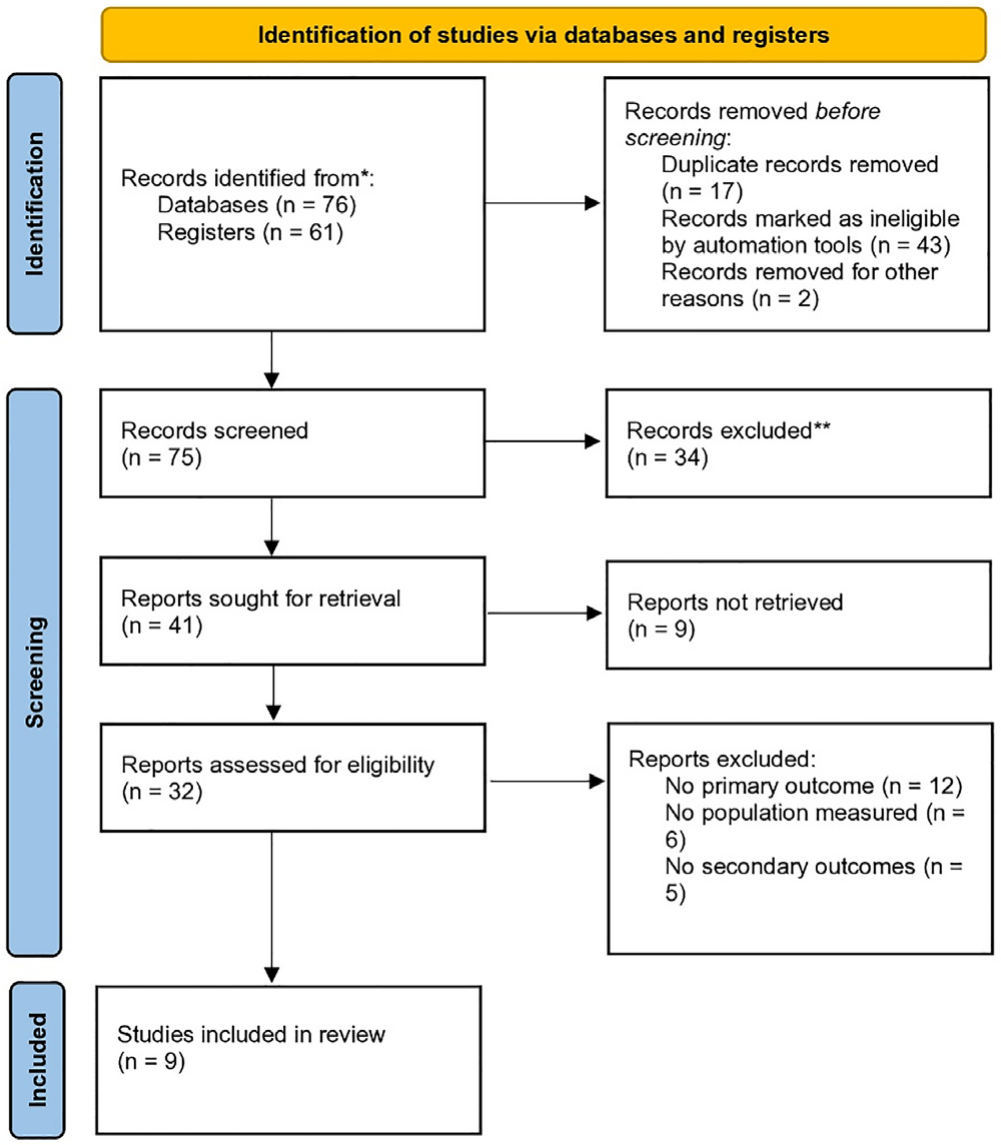
PRISMA flow chart. PRISMA = preferred reporting items for systematic reviews and meta-analyses.

This systematic review follows a structured and rigorous approach to evaluate the effectiveness and cost-efficiency of various CRC screening methods. The review adheres to preferred reporting items for systematic reviews and meta-analyses (PRISMA) guidelines (Fig. 1). The inclusion of both observational and interventional studies allows for a comprehensive analysis of screening methods across diverse populations.

### 2.2. Search strategy

A systematic search of electronic databases, including PubMed, Scopus, Cochrane Library, and Web of Science, was conducted to identify relevant studies. The search was limited to articles published between 2000 and 2024. Keywords and Medical Subject Headings (MeSH) terms included: “CRC screening,” “colonoscopy,” “FIT,” “CTC,” “sigmoidoscopy,” “screening effectiveness,” “cost-efficiency,” “risk populations,” and related terms. The search strategy was adjusted for each database to maximize sensitivity while minimizing bias.

### 2.3. Eligibility criteria

Studies were included based on the following criteria:

#### 2.3.1. Population

Individuals of any age with a focus on average-risk, high-risk (e.g., family history, age), and very high-risk (e.g., genetic conditions, inflammatory bowel disease) populations.

#### 2.3.2. Intervention

Studies comparing CRC screening methods, including colonoscopy, FIT, CTC, sigmoidoscopy, and stool-based DNA tests.

#### 2.3.3. Outcome measures

Effectiveness (e.g., sensitivity, specificity, detection rates) and cost-efficiency (e.g., cost per detected case, overall cost-benefit).

#### 2.3.4. Study design

Randomized controlled trials (RCTs), cohort studies, and cross-sectional studies.

### 2.4. Exclusion criteria

Studies were excluded based on the following:

Studies without a comparison of CRC screening methods. Articles not reporting on both effectiveness and cost-efficiency. Studies focusing on symptomatic populations or those with a prior history of CRC or colorectal polyps.

### 2.5. Screening and selection

Two independent reviewers screened the titles and abstracts of all identified articles to determine eligibility. Full-text articles were retrieved for further review. Disagreements between reviewers were resolved through consensus or by consulting a third reviewer. A detailed PRISMA flowchart was created to document the study selection process.

### 2.6. Data extraction

Data from eligible studies were independently extracted by 2 reviewers using a standardized data extraction form. Extracted information included study characteristics (author, year of publication), patient population details (age, risk category), type of screening method, effectiveness outcomes (e.g., sensitivity, specificity, detection rate), and cost-efficiency outcomes (e.g., cost per case detected, cost-effectiveness ratio). Any discrepancies in data extraction were resolved through discussion.

### 2.7. Quality assessment

The methodological quality of the included studies was assessed using the Cochrane risk of bias tool for RCTs and the Newcastle–Ottawa scale for observational studies. Each study was evaluated for risk of bias in domains such as selection bias, performance bias, detection bias, and reporting bias. The overall quality of evidence was graded as high, moderate, or low.

### 2.8. Data synthesis and analysis

A qualitative synthesis was performed to summarize the findings from included studies. Quantitative analysis, was conducted for comparable data regarding screening effectiveness and cost-efficiency, using random-effects models. Subgroup analyses were performed based on risk populations (average-risk, high-risk, very high-risk). Sensitivity analysis were conducted to assess the robustness of the results.

### 2.9. Software and interpretation

Statistical analysis was conducted using SPSS. The interpretation of results focused on the effectiveness and cost-efficiency of each screening method, considering both statistical significance and clinical relevance. The findings were discussed in the context of their applicability to clinical practice, particularly for different risk populations in the Table 1 mentioned below.

**Table 1:** Summary of the 9 mentioned articles.

Author name	Publication year	Screening methods	Effectiveness metrics	Cost efficiency	Population characteristics	Risk and limitations	Long-term outcome
Miliam P. van der meulen, Robert E Schoen et al^[1]^	2017	Fecal-immuno chemical test (FIT), micro simulation model (MISCAN-colon)	Women: 35.7 QALYs gained Men: 49.0 QALYs gained. Annual screens: Women: 8.7 QALYs Men: 6.7 QALYs	1. Biennial FIT screening: cost higher for women as compared to men. 2. Annual FIT screening: Women: 26,394 euro Men: 20,863 euro	Women and men aged 50–75 yr were studied. Data derived from FIT screening (CORERO-1) pilot study.	Women demonstrated lower test effectiveness than men in terms of QALYs gained. Gender stratified strategies yielded only marginal improvements in QALYs gained.	Optimal due to its comparable effectiveness and cost efficiency across gender. Provided at most 7% more QALYs but was not significantly more cost-effective.
Sujha subranium Florence et al^[4]^	2020	Providers and patient reminders. Provider assessment and feedback. Incentives.	Interventions resulted in a 4.9–26.7% point increase in CRC screening rates.	Incremental cost per person screened: Ranged from $18.6– $144.55 depending on the interventions and health system. Cost decreased for one awardee overtime due to reduce development and start-up costs	Population served by various health systems across United States, likely targeting individuals eligible for CRC screening ages 45–75 per guidelines.	Potential variability in implementation outcomes due to differences in health system capacities, demographics and patient’s adherence. Small sample size n = 9 awardees	Inhanced quality improvement, integration into training programs and data- driven programs redesign. Potential for broader adoption and cost effective interventions to sustain and increase CRC screening rates nationally, ultimately reduce morbidity and mortality
Sow-Neng Pang, Yu-Lun Lin et al	2022	1. Urinary micro RNAs (miR-21 and miR-141). 2. Fluorescein isothiocyanate for targeted sensing. 3. biotinylated probe.	Sensitivity: miR-21: Disease presence: *P* = .0176 Disease follow up: *P* = .00154 miR-141: Disease presence: *P* = .0032 Disease follow up: *P* < .0005 Specificity: Successfully differentiates disease presence, severity and progression.	Utilizing urinary samples minimizes patients discomfort and procedural Costs compared to colonoscopy. Modified SPCE reduces operational and production costs.	Focused on CRC patients at various stages of disease progression.	Risk of inaccurate results due to variability in miRNA expression. Validation across broader population and diverse demographics is required.	Offers a supplementary clinical tool for timely detection, monitoring and severity evaluation. Detecting miRNAs linked to cancer progression allows for early treatment and potentially improving survival rates.
Hidayati Hussaini Hasbullah, Marahaini Musa	2021	Gene therapy targeting p53 and KRAS mutation in CRC	Tumor suppressor encoding p53 (TP53). KRAS (oncogenesis). Improved survivability for CRC patients through targeted mutation repair. Enhanced specificity compared to conventional therapies.	High costs associated with developing and administering gene therapy.	Patients diagnosed with CRC, particularly those with advanced stage CRC harboring p53 and KRAS mutation.	Lack of widespread availability of gene therapy delivery systems. High costs, limiting accessibility in low resource settings. Ethical implications of gene editing and experimental treatments.	Improved survival and disease control but notes the uncertainty of long term outcomes due to limited clinical application data, unknown durability of genetic modification, potential for recurrence/ unforseen complications.
Shweta Ramesh Urra	2009	Antibody mediated targeting for CRC diagnosis and treatment	Utilizes monoclonal antibodies (mAbs) to selectively target antigens over expressed on Cancer cells while sparing normal tissues. Improved treatment selectivity. Reduced systematic toxicity compared to traditional chemotherapy.	Need for advancement in antibody therapy development because of its high specificity and reduced systematic toxicity compared to traditional chemotherapy.	CRC patients with tumors over expressing specific antigens that monoclonal antibodies can target.	Impaired tumor blood supply and lymphatic drainage, limiting therapeutic effectiveness. Poor infiltration and retention of antibodies with in tumor tissues. Limited efficacy against necrotic tissue and hypoxic regions of tumors. Development of resistance to antibody-based therapeutics. Potential for off-target effects or immune reaction.	The article emphasizes the potential of monoclonal antibody therapies to improve outcomes.
Sarah Cheuk Hei Chan, Jessie Qiaoyi Liang^[2]^	2022	FIT, Multi target stool DNA test, Stool bacterial and meta genomic markers, Fecal proteins, genetic and epigenetic markers. DNA methylation and mutation markers, Circulating tumor cells (CTCs), Micro RNAs, Imaging based modalities, Virtual colonoscopy, Colon capsule endoscopy.	Fecal biomarkers have better sensitivity than FIT for CRC. Gut microbiome and bacterial markers have promising diagnostic potential. Colonoscopy remains the most effective for both diagnosis and treatment but suffers from low patient compliance due to its invasive nature.	FIT is the most cost-effective, noninvasive test currently available. Virtual colonoscopy, mSEPT9 and multi target stool DNA tests are less cost-effective than FIT.	Adults age 45 and older or those with a high risk profile (i,e., family history, genetic predispositions.)	Lower sensitivity for early stage cancer and precancerous lesions compared to colonoscopy. Participation rates in stool-based tests and blood-based tests may still vary due to social, cultural or logistical factors.	Fecal biomarkers are expected to replace FIT as they become more accessible and cost-effective, offering high diagnostic accuracy. Early detection methods can significantly lower CRC mortality and incidence rates through timely interventions. Long term success depends on the affordability and global implementation of advanced screening technologies.
Sushmita barua	2023	AI-assisted colonoscopy. Standard colonoscopy	QALYs gained	AI-assisted colonoscopy is a cost effective strategy, offering the highest health gains at the lowest cost when initiated at age 50.	Hypothetical cohort of 100,000 men and women. Average risk of CRC. Ages at first invitation: 50, 55, and 60 yr.	Scenario analysis with 70% participation showed less favorable outcomes compared to the main analysis with 100% participation.	AI-assisted colonoscopy was cost-effective across all scenarios and implementation ages. Early implementation (age 50) of AI-assisted colonoscopy provided the highest health gains at the lower cost.
Joanne M. Hathway et al	2020	Colonoscopy (83% of the current screening mix). FIT (11% of the current screening mix). Multi target stool DNA (mt-sDNA) 6% of the current screening mix	Increased mt-sDNA utilization resulted in fewer Colonoscopies, lower adverse events and a slight increase in cancer detection, maintaining screening adherence rates over the 10 yr period.	Increasing mt-sDNA utilization for CRC screening leads to significant cost savings with integrated delivery networks (IDNs) saving $19.6 million and payers, saving $4.4 million over 10 yeas. These savings arise from reduced screening and surveillance Colonoscopies along with fewer adverse events.	Adults aged 50–75 yr with the analysis expanding eligibility to 45–75 yr old in one scenario. The analysis was based on a population of 1 million covered lines.	Direct non-medical costs (e.g, navigation) and indirect costs (e.g, administration) were applied per person, per modality though in reality some of these costs may be fixed. The inclusion of older adults (45–75 yr old) slightly reduced the overall cost savings.	Over a 10 yr period, increasing mt-sDNA utilization results in lower overall screening costs, improved cancer detection and fewer adverse events. The approach maintains screening adherence while reducing the needs for Colonoscopies.
MP- Van Der Meulen, I Lansdrop Vogelaar et al^[3]^	2018	CTC	Assessed using quality-adjusted life years (QALYs) gained. Colonoscopy (21.5%) CTC (33.6%)	Colonoscopy was more cost-effective in strategies with one or 2 life time screens. CTC was more cost-effective than colonoscopy for multiple lifetime screenings, especially with higher participation rates. However, colonoscopy was preferred if participation rates were the same for both methods.	Participants of this study study were enrolled in RCT. With optimal age range and interval for screening.	Excluded from the study due to lack of long-term follow-up data.	CTC screening for CRC is more cost-effective than colonoscopy due to its higher participation rates. However practical implementation requires careful consideration of handling extracolonic findings.

CRC = colorectal cancer, CTC = CT colonography, FIT = fecal immunochemical test, mt-sDNA = multi-target stool DNA, RCTs = randomized controlled trials, SPCE = screen-printed carbon electrode.

## 3. Results

A total of 60 articles were identified through database searching. After screening titles and abstracts, only 9 studies met the inclusion criteria and were included in this review (Table 1). These consisted of 3 RCTs, 2 cohort studies, 1 cross-sectional study, 2 retrospective studies, and 1 prospective study. The studies evaluated CRC screening methods across different risk populations, including average-risk, high-risk, and very high-risk individuals.

Various CRC screening modalities were assessed, including colonoscopy, FIT, CTC, sigmoidoscopy, and stool-based DNA tests such as multi-target stool DNA (mt-sDNA). The sensitivity of each method varied based on test type, cancer stage, and the risk profile of the population. Colonoscopy demonstrated the highest sensitivity – approximately 95% for detecting CRC and 90 to 95% for advanced adenomas – making it the gold standard, particularly for high- and very high-risk populations due to its ability to detect and remove precancerous lesions in a single procedure. FIT, a widely used noninvasive test, had a sensitivity of around 79 to 81% for CRC and 52 to 68% for advanced adenomas. Although less sensitive for adenomas, FIT performed well for CRC, especially in average-risk populations, and was particularly effective in detecting left-sided colon cancers.

CTC, also known as virtual colonoscopy, had a sensitivity of approximately 93 to 95% for CRC and 60 to 85% for advanced adenomas. While it offered a noninvasive alternative to colonoscopy, its lower sensitivity for smaller polyps was a limitation. Sigmoidoscopy had a lower overall sensitivity, around 60 to 70% for CRC detection, as it only examines the lower part of the colon (sigmoid colon and rectum), making it more suitable for limited screening in average-risk populations. Stool-based DNA tests, such as mt-sDNA, showed a sensitivity of about 85 to 92% for CRC and around 40 to 50% for advanced adenomas. These tests offered a convenient, noninvasive option for CRC detection and were particularly beneficial in increasing compliance among average-risk individuals. Overall, colonoscopy remained the most sensitive and comprehensive screening option, especially for high- and very high-risk populations, while FIT and mt-sDNA were more suitable for average-risk groups due to their cost-efficiency and non-invasiveness.

## 4. Discussion

This review highlights the critical role of test sensitivity, population risk, participation rates, and cost-effectiveness in shaping CRC screening strategies. Colonoscopy, due to its high sensitivity and ability to detect and remove lesions during the same procedure, remains the preferred screening tool for high- and very high-risk individuals.^[9]^ However, its invasive nature, higher cost, and lower participation rates limit its use in the general population. For average-risk populations, noninvasive tests such as FIT and mt-sDNA offer a balance between efficacy and acceptability, leading to higher compliance and broader screening coverage.^[1,10]^

Table 2 summarizes the comparative performance of CRC screening methods across different population risk groups. Among average-risk individuals, colonoscopy demonstrated the highest sensitivity for both CRC (~95%) and advanced adenomas (90–95%), though it was associated with moderate cost-effectiveness and lower participation rates.^[11]^ The FIT, while less sensitive for advanced adenomas (52–68%), offered strong performance for CRC detection (79–81%) and was noted for its high cost-effectiveness and participation.^[12]^ Similarly, stool-based DNA testing (mt-sDNA) showed strong sensitivity for CRC (85–92%) and moderate sensitivity for adenomas, with the added benefit of being noninvasive and widely acceptable.^[13]^ CTC also demonstrated high sensitivity for CRC (93–95%) but slightly lower for adenomas, and was more acceptable to patients, leading to higher participation. Sigmoidoscopy had the lowest sensitivity among these methods and was therefore considered supplementary, especially for distal colon examination.^[2]^

**Table 2:** Screening methods performance by risk group.

Risk group	Screening method	Sensitivity for CRC (%)	Sensitivity for advanced adenoma (%)	Cost-effectiveness	Participation rate
Average-risk					
	Colonoscopy	−95	90–95	Moderate	Low to moderate
	FIT	79–81	52–68	High	High
	CTC	93–95	60–85	Moderate to high	High
	Sigmoidoscopy	60–70	−50	Moderate	Moderate
	mt-sDNA	85–92	40–50	High	High
High-risk					
	Colonoscopy	−95	90–95	High	Moderate
	CTC	93–95	60–85	Moderate	High
	AI-assisted colonoscopy	>95	95+	High	Moderate
Very high-risk					
	Colonoscopy	−95	90–95	High	Low to moderate
	Sigmoidoscopy	60–70	−50	Low	Moderate

In high-risk populations, colonoscopy continued to be the most effective and clinically preferred method due to its ability to detect and remove pre-cancerous lesions.^[14]^ CTC remained a viable noninvasive alternative, particularly in settings with higher screening hesitancy. Notably, AI-assisted colonoscopy demonstrated enhanced sensitivity for both CRC and advanced adenomas, offering promise in improving outcomes and efficiency.^[15]^

**Figure 2.**
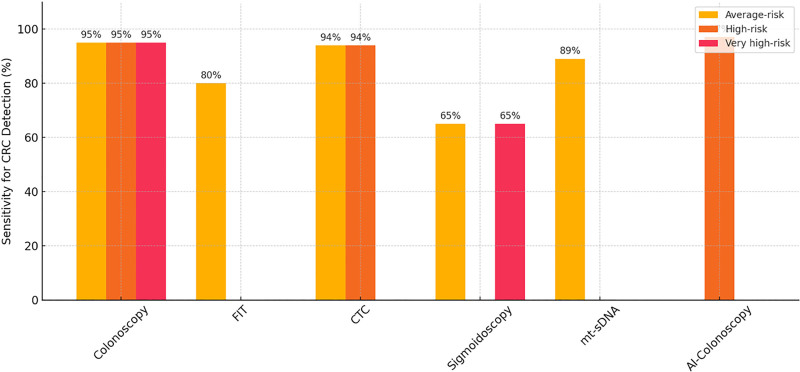
Sensitivity of CRC screening methods by risk group. CRC = colorectal cancer.

The Figure 2 bar chart compares the sensitivity of major CRC screening methods (Colonoscopy, FIT, CTC, Sigmoidoscopy, mt-sDNA, and AI-Colonoscopy) across average-risk, high-risk, and very high-risk populations. It visually emphasizes colonoscopy’s consistently high sensitivity across all risk groups and highlights the more limited applicability or performance of other methods in higher-risk groups.^[16]^

For very high-risk individuals, colonoscopy was clearly the gold standard due to its diagnostic accuracy and therapeutic capability. Sigmoidoscopy, while used in some cases, was markedly less effective due to its limited scope.^[17]^ Overall, the table illustrates that while noninvasive methods are better suited for average-risk populations due to ease of use and cost-efficiency, high- and very high-risk individuals benefit most from colonoscopy-based strategies due to their greater diagnostic demands.^[18]^

Participation rate is a major factor affecting cost-effectiveness. While colonoscopy is more cost-effective when performed less frequently, CTC may be preferable in settings where more frequent screening is required or where individuals have lower willingness-to-pay thresholds.^[4]^ However, current analyses often exclude extracolonic findings from CTC, which may underestimate its long-term value. Increasing use of mt-sDNA has shown promise in reducing overall screening-related costs and adverse events while improving detection and maintaining adherence over time. These benefits are especially relevant for healthcare systems and payer organizations aiming for cost-effective long-term strategies.^[19]^

**Figure 3.**
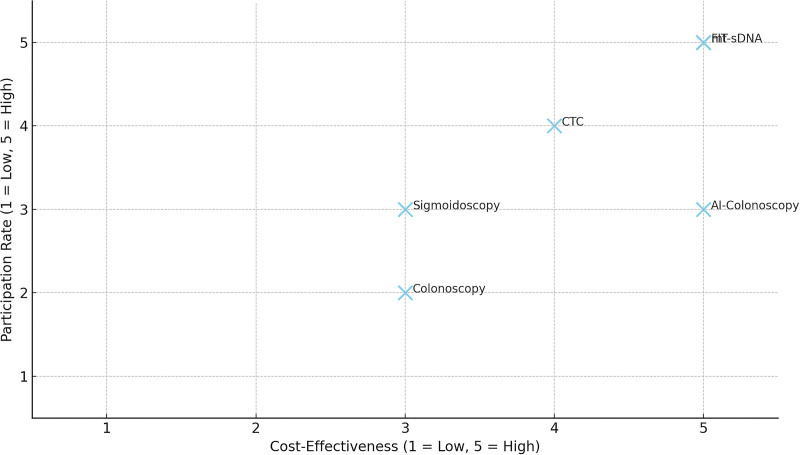
Cost-effectiveness vs participation rate of CRC screening methods. CRC = colorectal cancer.

Artificial intelligence (AI)-assisted colonoscopy is an emerging innovation with the potential to improve detection rates and reduce interval cancers. Studies suggest that initiating screening with AI-assisted colonoscopy at age 50 results in optimal health gains and cost-effectiveness, even when participation is suboptimal. Additionally, new biomarker-based approaches – including stool bacterial DNA, fecal proteins, and gut microbiome analysis – show potential for improving the diagnostic accuracy of noninvasive screening.^[20,21]^ Though still under investigation, these methods may address the current limitations of FIT and stool-DNA tests. The scatter plot maps in Figure 3 shows screening methods based on their cost-effectiveness (x-axis) and participation rate (y-axis). It clearly shows that noninvasive methods like FIT and mt-sDNA rank high on both axes, while colonoscopy, despite its high effectiveness, suffers from lower participation. AI-assisted colonoscopy holds potential for high effectiveness, though real-world participation data may still be evolving.^[22]^

Gender-specific differences in FIT performance have also been observed, with women demonstrating slightly lower sensitivity. However, analyses indicate that gender-based screening does not offer a significant cost-effectiveness advantage over uniform strategies.^[23]^ Behavioral interventions, such as personalized reminders and incentive-based programs, have also been shown to improve CRC screening uptake by up to 26.7% points, depending on the method and setting.^[24]^

Despite the promising results, this review is limited by the small number of included studies (n = 9), which affects the generalizability of the findings. Most of the available evidence was derived from average-risk populations, with relatively few studies focused on high- and very high-risk groups.^[25]^ Additionally, variations in methodology and outcome reporting across studies further limit the ability to draw broad conclusions. Future research should focus on long-term outcomes, real-world implementation, and the effectiveness of emerging technologies in diverse risk populations. Addressing these gaps is essential for optimizing CRC screening strategies and reducing the global burden of CRC.^[26,27]^

## 5. Conclusion

This systematic review compares the effectiveness and cost-efficiency of CRC screening methods across risk populations. Colonoscopy remains the most effective tool, particularly for high-risk groups, due to its ability to detect and remove precancerous lesions, significantly reducing cancer incidence and mortality.^[28]^ However, its invasiveness and preparation requirements often limit adherence.^[29]^ The FIT is a less invasive, cost-effective alternative for average-risk populations.^[30]^ Although less sensitive than colonoscopy, its simplicity, affordability, and higher acceptability make it ideal for large-scale screening. Annual FIT, combined with colonoscopy for positive results, strikes a balance between adherence and effectiveness.^[31]^ FIT is the most economical choice for average-risk individuals, while colonoscopy is cost-efficient for high-risk groups where advanced lesions are more common.^[32]^ In resource-limited settings, FIT or stool-based DNA tests offer practical, scalable solutions.^[33,34]^ Tailored, risk-based screening strategies are essential to maximize effectiveness and cost-efficiency, as a one-size-fits-all approach is suboptimal. Emerging technologies, such as artificial intelligence and biomarker-based diagnostics, hold promise for improving accuracy and refining screening practices in the future.^[35–37]^

## Author contributions

**Methodology:** Abida Parveen.

**Project administration:** Sadaf Showkat.

**Resources:** Corey Geoffrey Knowles, Abdul Khan Basit.

**Supervision:** Farukhzad Hafizyar, Sadaf Showkat, Asadullah Memon.

**Validation:** Asadullah Memon, Abida Parveen.

**Visualization:** Abdulrahman Atasi.

**Writing – original draft:** Abdul Subhan Talpur, Corey Geoffrey Knowles, Farukhzad Hafizyar, Abdul Khan Basit, Sadaf Showkat, Asadullah Memon, Abida Parveen.

**Writing – review & editing:** Abdul Subhan Talpur, Abdulrahman Atasi, Corey Geoffrey Knowles, Farukhzad Hafizyar, Abdul Khan Basit, Sadaf Showkat, Asadullah Memon, Abida Parveen.

## Correction

This article was originally published with an incorrect abstract. The incorrect abstract has now been corrected in the online version.
